# Vitamin D level, pain severity and quality of life among hemodialysis patients: a cross-sectional study

**DOI:** 10.1038/s41598-022-25793-z

**Published:** 2023-01-21

**Authors:** Shaima Ishtawi, Dana Jomaa, Aisha Nizar, Mazen Abdalla, Zakaria Hamdan, Zaher Nazzal

**Affiliations:** 1 Department of Medicine, Faculty of Medicine and Health Sciences, An-Najah National University, Nablus, Palestine; 2 Department of Orthopedics, An-Najah National University Hospital, Nablus, Palestine; 3 Internal Medicine Department, An-Najah National University Hospital, Box 7,707, Nablus, Palestine; 4 Department of Medicine, Faculty of Medicine, An-Najah National University, Box 7,707, Nablus, Palestine

**Keywords:** Medical research, Nephrology, Risk factors

## Abstract

This cross-sectional study aims to find the prevalence of chronic pain and its correlation with the quality of life and vitamin D levels among hemodialysis patients in Palestine. We used the brief pain inventory, the medical outcomes study 36-item short-form health survey, and Serum 25-hydroxyvitamin D to assess chronic pain, quality of life, and vitamin D levels, respectively. The study included 200 patients, 38.1% (95% confidence interval 31.3–45.4%) of whom had chronic pain, and 77.7% (95% confidence interval 71.0–83.4%) had deficient Vitamin D levels. Quality of life scores were generally low, with the lowest in role emotional and physical functioning. Sex, comorbidities, and vitamin D level significantly correlate with pain severity. Employment, number of comorbidities, pain severity, and albumin level are significantly associated with the Physical component of quality of life. On the other hand, employment and pain severity are significantly related to the mental component of quality of life. In conclusion, low vitamin D levels, chronic pain, and low quality of life scores are common among hemodialysis patients. In addition, vitamin D is negatively correlated with pain severity. Therefore, healthcare workers should assess and manage hemodialysis patients' chronic pain to improve their quality of life and reduce suffering.

## Introduction

Chronic kidney disease (CKD) is a common disabling disease with a prevalence of approximately 700 million people worldwide^1^. End-stage kidney disease (ESKD), the last stage of CKD, is an estimated glomerular filtration rate of less than 15 mL per minute per 1.73 m^2^ body surface area or those requiring dialysis^2^. The current management of ESKD is renal replacement therapy, either by dialysis or transplantation^3^. Saving the lives of ESKD patients is very important, and dialysis has succeeded in doing so, but maintaining a high quality of life (QoL) is also essential. Patient's quality of life may be impacted by hemodialysis due to its chronic nature, frequency, and the fact that most suffer from additional conditions that make them reliant on others. Quality of life, a general term for well-being, covers many aspects of individual life, including wealth, employment, environment, physical and mental health, and religious beliefs. The concept of health-related quality of life (HRQoL) has been used to correlate a people's health status to their QoL. It has been investigated among hemodialysis patients, and some determinants have been proposed, with the majority of scores indicating that it is decreased in these patients^4^. Demographic characteristics (e.g., age, educational level, income, and sex), and clinical and laboratory characteristics (e.g., number of comorbidities, medications, CKD complications, and hemoglobin < 11) have been found to correlate with HRQoL in hemodialysis patients^5^.

Pain is a common complaint among hemodialysis patients. Recent studies show that about 33%-82% of hemodialysis patients report chronic pain^6^. In contrast, the prevalence of chronic pain in the general adult population with or without kidney problems in the US was 20%^7^. Chronic pain could limit the daily activities of hemodialysis patients and affect their QoL^8^. Some social, clinical, and laboratory factors have been suggested as chronic pain determinants, including age, educational level, employment, body mass index and comorbidities, hemoglobin, and vitamin D^9^.

Vitamin D is a lipid-soluble vitamin that can be taken from the diet or synthesized in the skin and converted into its active form by a two-step process in the liver 25-hydroxylation and kidney 1-hydroxylation. Vitamin D deficiency, defined as the level of 25-hydroxyvitamin D (< 20 ng/mL)^10^, is an emerging worldwide problem in the general population^11^. In particular, ESKD patients are at high risk of vitamin D deficiency, with some studies finding the prevalence was 80%^12^. Furthermore, the role of vitamin D in chronic pain has been reported in some literature^13^. Again, its relation with QoL has been studied with controversial results; some noted that QoL's physical and mental components are affected, but others found that only the mental component is affected^14,15^.

In the Occupied Palestinian Territories, hemodialysis patients have increased in the last few years. The prevalence of vitamin D deficiency among hemodialysis patients was found to be approximately 87%^16^. Given the Palestinians' difficult living conditions and the unique circumstances of ESKD patients, we anticipated their QoL would be severely impacted. Some studies have investigated the QoL and its relation to chronic pain^17^. However, the role of vitamin D deficiency in chronic pain and QoL is still under-assessed.

In the last decades, the mortality among ESKD patients has decreased at the expense of their QoL. However, efforts are still being made to know the possible determinants of QoL for these patients and how to improve it. Therefore, this study aimed to assess the Vitamin D level of Palestinian hemodialysis patients and correlate it with their chronic pain and quality of life.

## Methods

### Study design and population

This cross-sectional study was conducted between October 2021 to February 2022 at the hemodialysis unit of An-Najah National University Hospital, Palestine. It is the region's largest dialysis center, receiving referred patients from the entire North West Bank of Palestine, with over 300 patients currently undergoing chronic hemodialysis. We included patients who were over the age of 18 and had been on hemodialysis for more than six months. Those who were critically ill, had cognitive dysfunction, or refused to complete the questionnaire or provide informed consent were excluded.

The sample size was calculated as n = [DEFF*Np (1 − p)]/ [(d2/Z21 − α/2*(N − 1) + p*(1 − p), with the confidence level set at 95%, the power set at 80%, the population size set at 2000, and the expected frequency of the outcome (Vitamin D deficiency) set at 70%, yielding 212 people. Patients were selected conveniently, and 224 agreed to participate, with 200 patients meeting the inclusion criteria and 24 being excluded; five were under 18, nine were on dialysis for less than six months, four were severely ill, and six had difficulty understanding the questionnaire.

The study and its associated experimental protocols, such as drawing blood, were approved by the An-Najah University Institutional Review Board [Reference #: Med. April 2021/14]. All procedures performed in this study have been carried out following the Declaration of Helsinki and relevant national guidelines and regulations. Patients were invited to participate in the study voluntarily after explaining the goal, objectives, and risk of involvement. No identifying information was collected, and patients were referred to as codes. Access to collected data was restricted to the study team and was used solely for research purposes. Informed consent was obtained from all patients.

### Study variables and measurements tools

Demographic, clinical, and laboratory data were collected from the patient's medical records. This included age, sex, body mass index, living arrangement, residency, marital status, educational level, employment status, income, smoking habits, dialysis duration, comorbidities (e.g., hypertension, diabetes mellitus, ischemic heart disease, stroke, peripheral artery disease, malignancy, liver disease, thyroid disease, and others), pain severity and its duration. The laboratory parameters were vitamin D level, parathyroid hormone, alkaline phosphatase, phosphorus, calcium, albumin, ferritin, and hemoglobin. In addition, serum 25-hydroxyvitamin D was measured at the beginning of the data collection period using the Elecsys vitamin D total test. Patients were classified as vitamin D deficient if their 25-hydroxyvitamin D level was less than 20 ng/ml and nondeficient if it was equal to or greater than 20 ng/ml^10^.

We used the brief pain inventory (BPI) scale to measure pain intensity and asked about the duration of pain. We considered pain for more than three months as chronic pain. The BPI, a widely used, valid, and reliable tool, is divided into two sections: one for pain intensity and one for pain interference with everyday activities. The pain severity scale was employed to determine the severity of the pain. It consists of four questions: worst, least, average, and current pain, each rated from 0 (no pain) to 10 (worst pain), with the mean of these four questions indicating the pain severity. Pain interference of daily activity scored as the mean of seven items (activities in general, mood, walking ability, sleep, work, interpersonal relationships, and taking opportunities in life). The pain was classified as mild (1–4 points), moderate (5–6 points), or severe (7–10 points). The Arabic version of the BPI was found to be reliable and valid for usage among Arabic-speaking patients^18^. Additionally, we computed Cronbach's alpha to determine the internal consistency of our tool, which was 0.91, suggesting excellent reliability.

The medical outcome study 36-item short-form health survey (SF-36) was used to assess the HRQoL. The SF-36 questionnaire assesses eight dimensions of HRQoL, yielding two summary measures: physical and mental health. The Physical Component Summary (PCS) includes four dimensions: physical functioning (10 questions), physical role (4 questions), bodily pain (2 questions), and general health (5 questions). The Mental Component Summary (MCS) includes four dimensions: vitality (3 questions), social functioning (4 questions), role emotional (5 questions), and mental health (2 questions)). The final question, self-reported health transition, is answered by the patients but not counted in the scoring process. The score of each dimension ranges from 0 to 100, with a higher score indicating better health. PCS and MCS scores are expressed by either the sum or the average of dimensions. The SF-36 has proven its reliability and validity^19^. In our study, Cronbach's alpha for PCS was 0.91 and for the MCS was 0.89, indicating excellent reliability. Before the beginning of the study, we conducted a pilot study of 15 patients to pretest the questionnaire and standardize the data collection method.

### Analysis plan

We conducted data entry, cleaning, and statistical analysis using IBM SPSS Statistics for Windows, version 21 (IBM Corp., Armonk, NY, USA). Descriptive analysis was used to describe the characteristics of patients using frequencies and percentages for categorical variables, mean ± standard deviation (SD) or medians, and interquartile ranges for continuous data. We used Cronbach’s alpha to check for the reliability of the used scales. The normality of the data was checked using the Kolmogorov–Smirnov test. We used the Mann–Whitney U and Kruskal-Walis tests to examine whether demographic and clinical variables are associated with MCS, PCS, and pain severity scale. Also, we used the Spearman correlation coefficient to test the correlation between patients’ Laboratory characteristics and their scores on the used scales. A multiple linear regression analysis model was used to determine the factors independently associated with QoL. All variables that demonstrated a significant relationship with pain and QoL in bivariate analysis were included in the model. A P-value of less than 0.05 was accepted as significant.

## Results

### Background characteristics

The study enrolled 200 hemodialysis patients, of whom 65% were males, and 54% were under the age of 60 years. A large proportion of the patients (79.9%) had only completed high school or below, 83% were unemployed, and 39.7% were smokers. A large number of patients (72.2%) have more than two years of dialysis. The number of comorbidities varies among patients, with 33% having more than four comorbidities. The results showed that 38.1% (95% confidence interval (CI) 31.3–45.4%) of hemodialysis patients had chronic pain, and 77.7% (95% CI 71.0–83.4%) had Vitamin D deficiency (Table 1).

**Table 1:** Demographic and clinical characteristics of the patients.

	Frequency* (%)	Median (IQR)
**Age in years**		59 (21)
≤ 60	108(54%)	
> 60	90(45%)	
**Sex**		
Male	130 (65%)	
Female	70 (35%)	
**Body mass index**		26.47(8.4)
Underweight	8 (4.2%)	
Normal	64(33.3%)	
Overweight	57(29.7%)	
Obese	63(32.8%)	
**living arrangement**		
Alone	13(6.7%)	
With family	181(93.3%)	
**Residency**		
Urban	92(47.4%)	
Rural	102(52.6%)	
**Marital status**		
Single	51(26.3%)	
Married	143(73.7%)	
**Educational level**		
School or less	155(79.9%)	
Collage or higher	39(20.1%)	
**Employment**		
Employed	33(17%)	
Unemployed	161(83%)	
**Income (US Dollar)**		
< 600	165(85.1%)	
600–1500	24(12.4%)	
> 1500	5(2.6%)	
**Smoking**		
Yes	77(39.7%)	
No	117(60.3%)	
**Comorbidities number**		
0	14(7%)	
1	29(14.5%)	
2	46(23%)	
3	39(19.5%)	
≥ 4	66(33%)	
**Duration of dialysis in months**		
≤ 2 years	54 (27.8%)	
More than two years	140 (72.2%)	
Patients with chronic pain	74 (38.1%)	
Patients with vitamin D deficiency	151 (77.7%)	

*Some variables don’t sum up to 200 due to missing data.

The QoL scores for different domains were calculated, with a higher score indicating better QoL. The average score of PCS was 41.4 ± 21.1, and the average score of MCS was 54 ± 24.4. We observed that the highest QoL score was in social functioning (67.1 ± 36.3), while the lowest was found in both role emotional (41.9 ± 45.9) and physical functioning (42.1 ± 32.3).

We conducted a bivariate analysis to find the possible factors related to pain severity and QoL main domains. Age and the number of comorbidities were related to pain severity, with a p-value equal to 0.037 and 0.003 for each, respectively. Sex, educational level, employment, number of comorbidities, duration of dialysis, and pain severity are significantly related to the PCS component of QoL. On the other hand, educational level, employment, number of comorbidities, duration of dialysis, and pain severity are significantly related to the MCS component of QoL (Table 2).

**Table 2:** Patients’ background and clinical characteristics with MCS, PCS, and Pain severity scores.

Characteristics	Pain severity Median (IQR)	P-value*	PCS Median (IQR)	P-value*	MCS Median (IQR)	P-value*
**Age**						
≤ 60 years	1.88 (4.6)	0.037	37.5 (21.2)	0.504	5.0 (41.8)	0.230
> 60 years	0.00 (4.2)		38.7 (28.2)		54.4 (39.3)	
**Sex**						
Male	0.00 (4.2)	0.093	43.7 (21.2)	0.002	52.0 (34.5)	0.568
Female	2.25 (5.2)		35.0 (20.0)		50.7 (32.2%)	
**Body mass index**						
Underweight	1.38 (5.8)		31.5 (17.9)		49.9 (46.1)	
Normal	0.00 (3.5)	0.572	38.7 (24.7)	0.341	55.4 (28.7)	0.427
Overweight	0.00 (4.8)		38.1 (31.0)		52.5 (27.1)	
Obese	0.75 (4.7)		37.9 (30.0)		46.1 (33.9)	
**living arrangement**						
Alone	2.5 (5.1)	0.279	32.5 (17.8)	0.154	46.0 (30.9)	0.943
With family	0.00 (3.7)		38.7 (30.3)		51.7 (41.2)	
**Residency**						
Urban	0.00 (4.1)	0.533	38.7(30.5)	0.332	58.6 (39.4)	0.112
Rural	0.00 (4.7)		37.5 (29.6)		48.9 (39.1)	
**Marital status**						
Single	0.00 (5.0)	0.493	37.5 (36.2)	0.897	49.3 (41.2)	0.796
Married	0.00 (3.2)		48.7 (31.2)		62.0 (33.0)	
**Educational level**						
High school or less	0.00 (4.7)	0.106	37.5 (26.2)	0.019	49.2 (41.0)	0.018
Collage or higher	0.00 (3.2)		48.8 (31.2)		62 (53.0)	
**Employment**						
Employed	0.00 (4.7)	0.167	58.8 (25.2)	< 0.001	78.0 (60.2)	0.007
Un- Employed	0.00 (2.8)		36.2 (27.2)		49.2 (34.3)	
**Income (Us Dollar)**						
< 600	0.00 (4.7)		37.5 (29.7)		51.1 (38.9)	
600–1500	0.00 (4.3)	0.758	41.5 (33.8)	0.205	66.4 (47.1)	0.230
> 1500	0.00 (4.6)		38.7 (53.1)		61.3 (39.0)	
**Smoking**						
Yes	0.00 (4.3)	0.750	40.6 (30.5)	0.204	47.2 (38.4)	0.132
No	0.00 (4.7)		37.5(35.6)		55.0(40.0)	
**Comorbidities number**						
0–3	0.00 (3.5)	0.003	45.3 (27.3)	< 0.001	55.5 (41.3)	0.005
≥ 4	1.5 (5.3)		31.2 (29.8)		46.6 (41.3)	
**Duration of Dialysis**						
≤ 2 years	0.00 (4.2)	0.274	39.4 (36.7)	0.068	61.3 (42.0)	0.070
More than two years	0.00 (4.7)		37.5 (39.7)		49.2 (38.3)	

*PCS* physical component summary, *MCS* mental component summary.

*Mann–Whitney U test and Kruskal–Wallis test.

Furthermore, we conducted a bivariate analysis to identify potential laboratory factors correlated with pain severity and QoL. The results showed that alkaline phosphatase, phosphorous, albumin, and vitamin D are significantly correlated with the PCS component of QoL. On the other hand, only alkaline phosphatase is significantly correlated with the MCS component (Table 3).

**Table 3:** Correlation between patients' Laboratory characteristics and their quality of life and pain severity scores.

Characteristics	Pain severity		PCS		MCS	
	Correlation coefficient	P-value*	Correlation coefficient	P-value*	Correlation coefficient	P-value*
Parathyroid hormone	0.052	0.475	0.039	0.585	− 0.049	0.498
Alkaline phosphatase	0.090	0.211	− 0.159	0.027	− 0.186	0.009
Phosphorus	− 0.016	0.821	0.193	0.007	0.118	0.103
Calcium	− 0.071	0.326	0.014	0.850	− 0.016	0.824
Albumin	0.065	0.367	0.202	0.005	0.043	0.549
Ferritin	− 0.003	0.966	− 0.087	0.228	− 0.016	0.827
Hemoglobin	0.017	0.819	− 0.003	0.970	0.012	0.870
Vitamin D	− 0.056	0.450	0.175	0.017	0.067	0.362
Pain severity	–	–	− 0.676	< 0.001	− 0.385	< 0.001

*PCS* physical component summary, *MCS* mental component summary.

*Spearman correlation test.

Multiple linear regression was run to find factors independently associated with pain severity and the QoL (Table 4). After adjustment, gender [P value = 0.011, B = 1.05, 95% CI 0.24–1.86], the number of comorbidities [P value = 0.011, B = 1.07, 95% CI 0.247–1.89], and Vitamin D [P value = 0.037, B = − 1.03, 95% CI − 0.061 to 1.99], were found to be associated with high pain severity. On the other hand, being employed, having a lower number of comorbidities, lower pain severity scores, and higher albumin level were associated with high PCS scores, whereas being employed and having lower pain severity scores were predictors of high MCS scores.

**Table 4:** Linear regression analysis for predictors of PCS and MCS domains of SF-36 and Pain severity.

Characteristics	Pain severity		Physical component summary			Mental component summary		
	B (95% CI) *	Adjusted P-value		B (95% CI) *	Adjusted P-value*		B (95% CI) *	Adjusted P-value*
Age	− 0.008 (− 0.036 to 0.020)	0.575		− 0.025 (− 0.196 to 0.145	0.770		0.20 (− 0.062 to 0.466)	0.133
Sex	1.05 (0.24 to 1.86)	0.011		− 1.72 (− 6.6 to 3.2)	0.493		3.3 (− 4.3 to 10.8)	0.394
Educational level	–	–		4.3 (− 0.123 to 9.7)	0.128		5.5 (− 3.1 to 14.1)	0.205
Employment	–	–		11.5 (4.8 to 18.2)	0.001		13.7 (3.3 to 24.1)	0.010
Comorbidities number	1.07 (0.247 to 1.89)	0.011		− 6.9 (− 11.6 to − 2.2)	0.005		− 257 (− 9.9 to 4.8)	0.492
Duration of dialysis	–	–		0.006 (− 0.041 to 0.053)	0.794		− 0.002 (− 0.77 to 0.072)	0.950
Pain Severity	–	–		− 4.49 (− 5.3 to − 3.6)	< 0.001		− 2.8(− 4.2 to − 1.6)	< 0.001
Alkaline phosphatase	–	–		0.00 (− 0.011 to 0.011)	0.958		0.004 (− 0.013 to 0.021)	0.629
Phosphorus	–	–		− 0.261 (− 2.1 to 1.59)	0.781		–	–
Albumin	–	–		12.6 (5.3 to 19.9)	0.001		–	–
Vitamin D	− 1.03 (− 1.99 to − 0.061)	0.037		0.084 (− 0.136 to − 0.305)	0.451		0.089 (− 0.243 to 0.421)	0.528

*PCS* physical component summary, *MCS* mental component summary.

*Unstandardized coefficients and its 95.0% confidence interval.

## Discussion

The present study showed low QoL scores, averaging 41.4 ± 21.1 for the PCS domain and 54 ± 24.4 for the MCS domain. This is consistent with previous studies^20–22^, indicating that the QoL in hemodialysis patients is lower than in the general population^4^. Additionally, the QoL was progressively impaired across the five CKD stages^5^. These findings could be attributed to the chronic nature of hemodialysis and possibly the patients' reliance on others, which affects them psychologically and their physical disease. This highlights the importance of developing methods for regular assessment and improvement of QoL.

Many studies have been conducted to find the factors that may affect QoL in hemodialysis patients. In the multivariate analysis, we found that employment, number of comorbidities, albumin level, and pain severity are all significantly related to QoL. Employment had a statistically significant positive effect on both PCS and MCS scores, similar to Samoudi AF et al.^17^. Others found no significant association^23,24^. Being employed allows hemodialysis patients to improve their social life and be more physically active, which may improve their QoL.

Furthermore, PCS was associated with increased comorbidities in this study, similar to a previous study in Palestine^17^. However, no association was found in studies conducted in Ethiopia and China^5,20^. Comorbidities exacerbate complications, resulting in increased patient complaints and decreased QoL.

Albumin levels correlated positively with PCS in this study. The same association was found in another research^4,23^. Protein-energy wasting may impair physical functioning in hemodialysis patients, so albumin levels may help assess those patients. On the other hand, we found that advanced age and income did not affect QoL, in contrast to other studies^17,24^. In many countries, ESKD patients are responsible for paying all or a portion of their dialysis treatment costs on their own, whereas hemodialysis patients in Palestine are provided with all health services for free; thus, income may appear to have a less impact on hemodialysis patients' QoL.

In our study, more than one-third of hemodialysis patients have chronic pain, similar to the prevalence in a Chinese study^20^. However, a systemic review and a previous study in Palestine reported a higher prevalence of chronic pain, 60.5% and 66.3%, respectively^9,25^. This difference could be due to different sample sizes or differences in pain perception among different cultures. Nevertheless, this indicates that pain is a prevalent complaint among hemodialysis patients and should be assessed routinely during their follow-up care.

Among the pain group, the median pain severity was 4.75, classified as moderate severity according to the BPI scale. Furthermore, we found that the severity of pain was negatively correlated with PCS and MCS scores in multiple regression analysis, which is consistent with the findings of other studies^8,17^. However, others found no association between pain severity and QoL^20^. Chronic pain patients often have physical limitations that make it more difficult for them to engage in daily activities and social interactions than healthy people, lowering their QoL. Furthermore, they occasionally deal with comorbid conditions such as anxiety, depression, and sleep problems, which reduce their QoL. This highlights the need for greater attention to pain and an early management protocol to reduce its possible negative effect on QoL.

Awareness of factors affecting chronic pain and its severity may facilitate pain management. Females have a higher pain severity than males in this study which is consistent with other studies^17,26^. Pain sensitivity is suggested to be higher in females, and their response to pain management could be lower^27^. Also, higher rates of depression and stress among females could be a contributing factor^28^. An increasing number of comorbidities is usually accompanied by increasing disease complications and patient suffering, which can be reflected as somatic pain. Our study shows a positive correlation between the number of comorbidities and pain severity; these results align with other literature^9,26,29^.

Vitamin D is a fat-soluble vitamin obtained primarily through sunlight-induced skin synthesis, with the remainder obtained through diet. The kidney is essential for vitamin D metabolism. Patients with ESKD are at increased risk for vitamin D deficiency because they frequently lack access to sunlight and adequate nutrition, and their impaired kidneys inefficiently reabsorb 25-hydroxyvitamin D. The prevalence of Vitamin D deficiency in our group is 77.7%, which is similar to the literature^16^.

Vitamin D is essential for musculoskeletal function because it aids in bone formation, maintenance, remodeling, and muscle protein synthesis. A lack of vitamin D increases bone resorption, resulting in osteomalacia, characterized by bone pain and muscle weakness, gait instability, recurrent falls, and fractures^30^. Another study suggests a potential interaction between vitamin D and its receptors with pain signaling genes and pathways; Vitamin D receptors have been identified in the brain, spinal cord, and dorsal sensory ganglia^31^. Our findings revealed a negative correlation between pain severity and vitamin D level, consistent with the results of a previous study^13^, whereas another study found a marginal correlation^32^.

On the other hand, some researchers suggest that vitamin D may play a role in neuropsychiatric function. For instance, people with low levels of 25-hydroxyvitamin D commonly have depression and score poorly on the mini-mental state test^33^. Additionally, vitamin D deficiency has been linked to CKD progression and increases morbidity and mortality in CKD and hemodialysis patients, potentially affecting QoL^34,35^. The role of vitamin D in QoL has been investigated here, but no significant correlation was found. In contrast, one study demonstrated the significance of vitamin D levels on PCS and MCS scores^14^, while another found the significance on MCS only^15^. The difference in measurement tools could explain this controversy, emphasizing the need for more research to clarify the possible relationship between vitamin D and QoL.

Some interventional studies demonstrated the possible beneficial effect of vitamin D supplementation among vitamin D deficient people in contrast to vitamin D sufficient people who showed no improvement in pain^36^. However, the evidence is still weak and needs more randomized control trials to put general recommendations about vitamin D supplementation. This study paves the way for additional investigation into whether vitamin D supplementation alleviates pain in these patients so that it might be routinely utilized in pain management.

This study has possible limitations. First, the cross-sectional study design limits the ability to suggest causality between related variables. Second, the results should be cautiously generalized because the study was conducted at a single clinical center. Thirdly, we did not gather information on certain psychosocial factors that may influence pain perception and QoL. Lastly, the quantitative nature of the data prevented a complete explanation of the reasons for the low quality of life from the patient's point of view, which would have been better revealed through in-depth interviews or focus group discussions.

In conclusion, our study demonstrated that low vitamin D levels and chronic pain are common among hemodialysis patients. Vitamin D level is negatively correlated with pain severity. Lower QoL scores were found in hemodialysis patients and found to be significantly related to unemployment, comorbidities, severe pain, and low level of albumin. Healthcare workers should devote more time to assessing and managing chronic pain in hemodialysis patients to improve their QoL and reduce their suffering. Further studies should be conducted to investigate more determinants of chronic pain and QoL, ways to assess and manage chronic pain and study the effect of some supplementations like Vitamin D on chronic pain and QoL.

## Data Availability

The dataset supporting the conclusions of this article are included within the article’s additional files.
